# Breaking bonds: maternal and offspring states relate to constraint-based brood donation in a seaduck

**DOI:** 10.1093/beheco/araf134

**Published:** 2025-11-21

**Authors:** Markus Öst, Benjamin Steele, Kim Jaatinen

**Affiliations:** Environmental and Marine Biology, Åbo Akademi University, Henriksgatan 2, 20500 Turku, Finland; Department of Bioeconomy, Novia University of Applied Sciences, Raseborgsvägen 9, 10600 Ekenäs, Finland; School of Arts and Sciences, Colby-Sawyer College, 541 Main Street, New London, NH 03257, United States; Nature Solutions, Finnish Environment Institute, Latokartanonkaari 11, 00790 Helsinki, Finland; Tvärminne Zoological Station, University of Helsinki, J.A. Palménintie 260, 10900 Hanko, Finland

**Keywords:** alloparental care, body condition, brood amalgamation, common eider, parental care, relative brain size, salvage strategy

## Abstract

The adaptiveness of alloparenting for donors, recipients and both natal and transferred offspring remains unsettled despite long-standing interest. Using decade-long data on individually marked female and duckling common eiders (*Somateria mollissima*), which frequently transfer offspring between broods, we examined factors influencing the likelihood of females donating young and ducklings being adopted. We explored how donor traits, including maternal body condition, relative head size (a validated proxy of relative brain size, potentially associated with risk assessment and reproductive decision-making) and relative hatching date, and offspring characteristics such as body condition relative to siblings, relate to these processes. At least one offspring was permanently adopted in 34.7% of brood observations. Females in better body condition and larger relative head size were less likely to donate offspring, while offspring transfer was more likely from larger natal broods. Offspring donation was most likely just before the population's hatching peak, suggesting that recipient availability influences adoption. Ducklings in poorer body condition than their natal broodmates and those whose mothers were in lower body condition were, respectively, significantly and marginally significantly more likely to be adopted. Taken together, duckling transfer is associated with physical and cognition-related characteristics of donors and adoptees, without necessarily implying an adaptive strategy for either. Multiple tending females per brood prevented assignment of adopted ducklings to a unique recipient; nonetheless, previous studies suggest recipient females may accrue fitness benefits. Future research quantifying the fitness consequences for all parties in different environmental contexts is required for a more comprehensive understanding of alloparental behavior.

## Teaser

Duckling transfer in eiders relates to maternal and offspring physical and cognitive limits and is not necessarily adaptive. Our study shows that mothers in good condition or with bigger heads (proxy for brain size) get to keep ducklings more often, while weaker ducklings and those in larger broods are more often adopted. Adoptions are also more common near peak hatching, when more caregivers are available. Brood donation functions as a salvage response under intrinsic constraints.

## Introduction

Alloparental care—care for non-descendant offspring—is the net result of the evolutionary interests of donors and recipients, which do not necessarily align with each other (Riedman 1982; Eadie et al. 1988; Wisenden 1999). Adoption and brood amalgamation have been documented in over 120 mammalian and 150 avian species (Riedman 1982). In waterfowl, post-hatch brood amalgamation is particularly common, occurring in at least 40 species (Eadie et al. 1988; Kalmbach 2006). For example, up to half of Canada goose (*Branta canadensis*) goslings may be raised in multi-family “gang” broods (Conover 2009). These findings demonstrate that postnatal offspring mixing is not merely anecdotal but a significant component of the reproductive biology of many vertebrates. However, despite its widespread occurrence, the causes and mechanisms of alloparental care are not well characterized in an intraspecific context (Yom-Tov 1980; Riedman 1982; Wisenden 1999; Tallamy 2005). This knowledge gap reflects, in part, the formidable challenge of identifying and tracking individual young during offspring transfer between donors and recipients, which often occurs rapidly and outside the confines of the nest or burrow (Kalmbach 2006).

Donors and recipients of young are likely to experience differential fitness outcomes. Caring for non-descendant offspring may raise the reproductive costs of recipients (Sorenson 1991; Lyon 2003). However, negative effects on recipient fitness may be mitigated if young are precocial (Andersson and Eriksson 1982; Tallamy 2005; Jaatinen et al. 2011) or if mechanisms such as predator dilution are at play (Öst et al. 2008a; Lehtonen and Jaatinen 2016). Recipients may even reap benefits at the donors' expense through selfish herding (Nudds 1980; Eadie and Lumsden 1985; Öst and Bäck 2003), which may impose a disproportionate predation risk on the adoptees (Nastase and Sherry 1997). These dynamics introduce two kinds of latent costs on prospective donors: direct fitness loss when offspring are transferred to other broods—a cost that may be exacerbated if adoptees are mistreated in the host brood (eg, Nastase and Sherry 1997; Öst and Bäck 2003)—and the energetic, survival or opportunity costs incurred when parents attempt to defend, retrieve, or maintain all offspring. The balance between these two types of costs should determine the direction of selection: when the cost of caring for larger broods is low (as is likely in precocial, self-feeding waterfowl), selection should favor parental traits that preserve brood integrity and offspring traits that promote a close physical connection to their natal brood (unless the mother completely abandons them).

Whereas offspring abandonment might ultimately serve a salvage strategy when opportunities for independent breeding are limited (Eadie et al. 1988; Lyon and Eadie 2008), the proximate underpinnings of this behavior are insufficiently understood. From a mechanistic perspective, poor-quality parents may be more prone to losing their young (Savard 1987), with parents actively attempting to prevent brood loss (van Breukelen and Santangelo 2020). The follow-up question is whether inexperienced donors, or those facing energetic constraints or cognitive demands, struggle more to maintain brood integrity, a task that may, for instance, require numerical competence (Lyon 2003). From the offspring's perspective, movement away from the natal brood can arise from proximate cues rather than any active preference. For example, ducklings may be attracted to nearby females that are actively brooding, more aggressive or dominant (Munro and Bédard 1977; Kalmbach 2006; Öst et al. 2007b; Wong and Kölliker 2013), whereas low-condition individuals may occupy peripheral positions because of reduced locomotor performance or starvation-related shifts in foraging behavior (Swennen 1989; Anderson and Alisauskas 2001; Krause and Ruxton 2002; Morrell and Romey 2008), making them more likely to become separated. It should be noted that these cues are not mutually exclusive: a poor-condition duckling at the edge of the group may be especially prone to orient toward a strong brooding stimulus and thus end up in a foster brood. Such cue-driven movements may lead ducklings to join broods providing higher-quality care or resources, without implying any active preference, yet potentially yielding fitness benefits.

To address these open questions about the donor and offspring sides of the adoption dynamics, we capitalized on a decade of data on a total of 2,668 individually marked ducklings in 689 broods belonging to 438 unique female common eiders (*Somateria mollissima*; hereafter, eiders) from the northern Baltic Sea. Eiders display communal brood care with frequent exchange of offspring (Öst et al. 2003a) and remain close to their breeding islands following nest exodus (Öst and Kilpi 2000), allowing us to study the process of offspring transfer. We note here that classical explanations for adoption and brood amalgamation include kin selection, reciprocal altruism, practice for parenting, and misdirected care/reproductive error (Pierotti 1980, 1982; Waltz 1981; Andersson and Eriksson 1982; Riedman 1982; Friesen et al. 1996; Brown 1998), but as the bulk of these hypotheses pertain primarily to recipient behavior, they are only acknowledged here while the present study focuses on donor mothers and transferred offspring. The term “donor’ is used as a neutral descriptor for the biological mother from whose brood one or more ducklings became permanently transferred, without implying intentionality on the part of the donor female.

Female reproductive strategies in eiders, a facultatively social species with uniparental female care, range from solitary to cooperative brood care—with group sizes tending to increase as predation risk rises and body condition declines—to complete brood abandonment when in poor body condition (Kilpi et al. 2001; Öst et al. 2003a, 2003b; Jaatinen et al. 2011). Ducklings that become separated from their brood are frequently adopted, yet they may experience nepotistic aggression from foster females, often ending up in more peripheral and dangerous positions within the brood (Öst and Bäck 2003). In this system, the speed of coalition formation is a key antipredator tactic, because prolonged searching for females willing to share brood-rearing duties increases exposure to undiluted predation risk. Females prefer female coalition partners with similar brood sizes: those with more similar brood sizes reach cooperative “agreements’ more rapidly than females with disparate brood sizes, reflecting negotiation over relative contributions to predator dilution (Jaatinen and Öst 2013). Importantly, coalition formation speed also varies with annual predation risk and female traits: relatively large-headed females—a trait closely correlated with brain size (Jaatinen et al. 2019)—form coalitions faster in dangerous years but more slowly in benign years, whereas small-headed females show no such adjustment (Öst and Jaatinen 2015). Relatively large-headed females also show a more pronounced breeding delay than their smaller-headed counterparts when dispersing to unfamiliar—and hence potentially more dangerous—nesting areas (Jaatinen and Öst 2016). Although direct tests of head-size-dependent cognitive performance are unavailable in this system, the observed patterns are compatible with greater sensitivity to perceived predation risk and with the possibility that relative head size is linked to the ability or tendency to assess predation risk and to modify brood-care strategies accordingly. Admittedly, these observational findings cannot in themselves establish causation between head size and cognitive abilities, but parallel experimental findings in guppies (*Poecilia reticulata*) offer supporting evidence, showing that larger-brained individuals exhibit enhanced predator inspection and spatial memory under predation threat (Burns and Rodd 2008; Kotrschal et al. 2013, 2015). Endocrine state may also contribute to individual variation in parental strategies in female eiders: elevated levels of baseline corticosterone have been associated with greater reproductive investment (Mohring et al. 2023), whereas higher prolactin levels appear to enhance reproductive success under constraining conditions (Mohring et al. 2024).

We focused on the donor's perspective because it was not possible to identify recipients unambiguously. In most broods, multiple brood-tending females are present, and we also lacked systematic observations of the initial contact between prospective adoptees and foster broods. Recipient behavior and traits are therefore not tested here and remain an important direction for future work. First, we compared the characteristics of donor females against a reference group of successful breeders that did not permanently transfer or lose any offspring (see Methods). The ability to maintain brood integrity may depend on parental body condition—eg due to condition-dependent vigilance effort (Öst et al. 2007a) or parental age—eg due to age-dependent parent-offspring recognition (Langmore et al. 2009). A third as-yet untested factor that may influence this ability is relative head size (used here as a proxy for relative brain size)—eg because ensuring that a brood of highly mobile young is complete may require cognitive abilities such as numerical competence (Lyon 2003), although this has not been tested in this system (see Methods). We therefore predicted that females in (1) good body condition, (2) older and more experienced, and/or (3) with relatively larger heads would be less likely to become “donors’. In a second analysis, we compared the body condition and size of fostered ducklings against their siblings remaining in the original brood, after controlling for maternal age and body condition, which may affect the strength of mother-offspring bonds (Kalmbach 2006). If all ducklings are similarly attracted to more broody females, the net bias among adoptees will be set by what happens after joining: higher-quality ducklings may outcompete conspecifics for parental care and/or survive better once adopted (cf. Brown 1998), producing an apparent excess of higher-quality adoptees. By contrast, a splitting driven by physical constraints (reduced locomotion or peripheral position) predicts only an excess of lower-quality adoptees. Because nesting density may pose a socio-ecological constraint on brood amalgamation by influencing encounter rates between broods and the availability of potential recipient females (Eadie et al. 1988), we controlled for seasonal variation in the availability of potential hosts in both analyses using the difference between each focal female's hatch date and the median annual hatch date (Jaatinen and Öst 2013).

## Material and methods

### Study area and female handling

The study was done in the Tvärminne archipelago (59°50′N, 23°15′E), western Gulf of Finland, from 2008 to 2017. Eiders breed on both small, open granitic islets (mean size ± SD = 0.37 ± 0.23 ha, *n* = 13) with patches of grass and occasional juniper (*Juniperus communis*) scrub, and larger islands (3.68 ± 3.22 ha, *n* = 9) with pine-dominated forest (*Pinus sylvestris*) with interspersed juniper shrubs. The study area is protected through a prohibition on landing except for research purposes. Handling of birds was approved by the Finnish Project Authorization Board of the Regional Administrative Agency for Southern Finland (permit numbers ESLH-2009-02969/Ym-23, ESAVI/1697/04.10.03/2012, and ESAVI/2831/04.10.07/2015), birds were ringed under a valid ringing license, and the study complied with the regulations of Tvärminne Zoological Station.

We captured incubating female eiders with hand nets during the later stages of incubation to minimize disturbance (Bolduc and Guillemette 2003). Each female was weighed to the nearest 10 g, measured for the length of the radius-ulna to the nearest 1 mm, ringed with a standard steel ring and individually coded color rings, and equipped with a uniquely colored temporary wing flag. Post-departure ringing of ducklings is impossible in this precocial species, and high juvenile mortality limits local recruitment; thus, only adults are ringed. The number of years since the bird was first ringed yielded a minimum age estimate (Öst and Steele 2010). This is a valid proxy because, first, the majority of successfully breeding females are annually captured, with a relatively constant annual capture effort: we captured on average 232.6 ± 16.8 successful breeding females per year (mean ± SE, n = 10), which corresponds to 62.8% ± 1.6% of the mean annual number of successful breeders (368.8 ± 22.5). Second, females show strong breeding philopatry at the island level (Öst et al. 2011). Hatch date was estimated using egg flotation, which infers embryo age from how eggs sink or float in water as their specific gravity changes during incubation; this method has been validated for our study species and population (Kilpi and Lindström 1997). Global body condition indices were determined for all captured females (*N* = 1,853, annual range 116 to 249 females) that had been incubating eggs for at least 8 days to ensure clutch completion (Öst et al. 2008b). This index was calculated as the standardized residuals of a regression of log-transformed projected weight at hatching (response variable) (see below) on log-transformed radius-ulna length (explanatory variable), and indices were derived for the pooled data from all years to allow between-year comparisons. We obtained estimated female weight at hatching by subtracting an estimate of the expected weight loss during the remaining incubation time from the measured incubation weight. Females were weighed once, but as females fast during incubation and we captured them at variable incubation stages, an estimate of average weight loss rate during incubation could be derived as the slope of the regression of log-transformed body weight (response variable) on log-transformed incubation time and projected hatching date (Öst et al. 2008b). The assumption of continued weight loss after female capture is valid in this study population (Öst and Steele 2010), and thus this body condition index provides comparable estimates of individual body reserves.

Head volume, which is strongly associated with brain mass in female eiders (*r*^2^ = 0.73; Jaatinen et al. 2019), was determined by multiplying head length, width, and height, measured with a precision of 0.01 mm. Measurements of head volume from a given year are representative of an individual's head volume in previous years. This is supported by a repeatability analysis based on an extended dataset spanning 2012 to 2022, which showed high within-individual repeatability across years (r ± SE = 0.71 ± 0.02, 95% CI = [0.67, 0.75], *P* < 0.001; rptR package; Stoffel et al. 2017). Head size measurements began in 2012, and this repeatability allowed us to include years with other data even when direct head volume measurements were unavailable (2008 to 2011). We determined relative head volume to account for the fact that head size tends to increase with body size. To this end, we calculated the standardized residuals from a linear regression where log-transformed head volume (response variable) was explained by log-transformed radius-ulna length (explanatory variable). Mean head volumes and radius-ulna lengths were used in this calculation if multiple measurements were available.

### Viable brood size, duckling handling and individual identification

Hatching is synchronous in eiders and ducklings leave the nest within 24 h of hatching (Öst and Bäck 2003). Handling of ducklings and the determination of viable brood size at hatching were usually done during nest revisits on the estimated hatching date determined by egg flotation (see above), but in rare cases immediately upon female capture (if the female was captured when the brood had just hatched). Hatching success was assessed by counting the number of viable offspring, or by inspecting the nest contents if the brood had already hatched and left the nest or it had been depredated. Hatched eggs have an intact leathery membrane, while depredated eggs leave shattered shells with the membrane, usually bloody, still attached to the shells (Öst and Steele 2010). To determine viable brood size, the number of unhatched or depredated eggs and dead or dying ducklings was subtracted either from the number of live ducklings found in the nest (if the brood was handled) or from the clutch size at the time of female capture (if signs of successful hatching indicated the brood had left the nest). If the brood had already hatched and left the nest and the nest was empty with no signs of predation, the viable brood size was deemed the same as the clutch size recorded at female capture (Öst et al. 2007a; Jaatinen and Öst 2013); this situation applied to 734 of 2,646 documented nest fates during the study period (27.7%). Although eggs may have been carried away by predators between the brood's leaving and our arrival at the nest, this potential bias is likely negligible, considering the short time interval (usually less than 24 h) and because eggs are typically eaten in the nest surroundings, leaving plenty of broken eggshells.

Each dry duckling in the nest was weighed to the nearest 1 g and measured for tarsus length to the nearest 1 mm. Ducklings were then marked with colored 2 × 1 cm pieces of plastic electrical tape, attached to the tip of nape feathers with cyanoacrylate super glue (Bustnes and Erikstad 1991), which lasts ca 2 weeks (Öst and Bäck 2003). The nape tags had an individual and a brood-specific color combination. The color palette consisted of eight colors, with a maximum of three colors per tag. Totally 2,668 ducklings in 689 broods belonging to 438 unique female common eiders were marked over the study (annual mean ± SD = 267 ± 57 ducklings, range 171 to 329). The large number of marked ducklings and the limited number of marking permutations resulted in some non-unique combinations. To alleviate this problem, we used identical marking combinations at different times of the breeding season and/or at distant locations. Nevertheless, in case of any ambiguity concerning the duckling's identity (ie, when at least two likely candidates remained), such observations were discarded from further analysis.

Individually marked females and young were observed and identified daily from suitable vantage points by a team of two to five observers equipped with binoculars and spotting scopes (20 to 70 ×). When a brood with at least one marked duckling was observed, we recorded the total number of marked and unmarked ducklings and females, and their identity. Eider brood-rearing coalitions show slight variations in size during the first two weeks post-hatch, attributed to the presence of females transiently joining the brood, as well as non-tending females joining and then leaving the brood within minutes, and being treated aggressively by the coalition members (Öst et al. 2003a, 2003b; Jaatinen et al. 2012). To ensure correct identification of the composition of the brood, we observed each focal brood for up to an hour or more. Because broods sometimes moved out of view and visibility occasionally deteriorated unpredictably (fog, strong backlight), it was not always possible to confirm the identity of every duckling in the field; all observations were subsequently consolidated and reviewed by a single experienced assessor (M.Ö.), who reconciled ambiguous identifications and identified offspring transfers, and unresolved records were excluded from analyses.

Because the availability of potential female coalition partners affects the time it takes to form brood-rearing coalitions (Jaatinen and Öst 2013), it may also affect the likelihood of duckling transfer between broods. The availability of potential recipients for each prospective donor female was approximated by the difference in absolute number of days between the focal female's hatch date and the annual median hatch date. The median hatch date was calculated on the basis of all females that successfully hatched a brood in each year. We expect the pool of available potential recipient females to be the greatest at peak hatching, while prospective donors hatching their broods before or after most other females will have fewer potential recipients available (Jaatinen and Öst 2013).

### Statistical analysis

The statistical analyses were run in R 4.0.4 (http://www.R-project.org/). We conducted two logistic regression analyses (GLM, binomial distribution with a logit-link function) to explore the factors that influence the likelihood of (1) females donating offspring to others, (2) ducklings ending up as adoptees. Importantly, untended ducklings are rapidly preyed upon and therefore lost but unadopted ducklings cannot be reliably documented; consequently, our analyses concern observed brood amalgamations only. In the first analysis, the response variable was coded as 1 if at least one offspring left its natal brood to join another brood not attended by the mother, and 0 if the mother successfully hatched a brood observed at sea with no offspring transfers. We only considered instances of permanent offspring transfer, ie, the duckling(s) concerned did not return to their natal brood at any subsequent time. In the second analysis, we focused on cases where at least one duckling from the brood was permanently incorporated into another brood, which did not include the mother. The response variable indicated whether the duckling moved to a new brood (1) or remained in its original brood (0). Importantly, only broods in which at least one duckling left its natal brood were included. This restriction was necessary to allow comparisons between adopted and non-adopted ducklings within the same brood. Because this analysis focuses on individual ducklings, this inclusion criterion creates a simple arithmetic effect: if only one duckling leaves, it represents a large fraction of a small brood (eg, 1 out of 2 = 50%), but a much smaller fraction of a large brood (eg, 1 out of 6 ≈ 17%). This means that, even if the number of ducklings leaving is the same, the per-duckling probability of adoption will inevitably decline with increasing brood size. This is a design-related effect and does not contradict the first analysis.

Explanatory variables included in both analyses were the donor female's age, body condition, viable brood size and the deviation between the focal female's hatch date and the annual median hatching date. This deviation was expressed as the linear and quadratic terms of the difference between the focal donor's hatch date and the annual median date, to capture any non-linearity in this relationship. Maternal relative head size was included only in the first model because initial testing showed it not to be associated with whether a duckling left its native brood, and thus this variable was omitted from the second model to maximize sample size and hence statistical power (brain size measurements started in 2012, see above). In the second analysis, offspring morphology—body condition, weight and tarsus length—was expressed relative to their siblings by mean-centering measurements within broods. The mean-centering approach was also chosen because duckling body condition at hatching shows considerable within-brood repeatability (r = 0.56, Öst et al. 2020). Repeatability here is the intra-class correlation (proportion of variance between broods), and thus r = 0.56 indicates that a majority of variation in condition is between broods; mean-centering therefore isolates within-brood deviations (individuals in poorer/better body condition than their siblings). Of these three collinear variables, we selected the most parsimonious one based on the best-performing single-predictor model based on Akaike's Information Criterion corrected for small sample size (AICc).

The first logistic regression model was based on 343 observations of 233 unique female eiders (female-level analysis) and the second one on 619 ducklings from 144 broods belonging to 124 unique females (duckling-level analysis), with both analyses spanning ten years. To test whether this hierarchical design causes pseudoreplication necessitating the use of random-effects models, we tested whether the variance components for female identity and year differed from zero. Because brood and female identity is effectively collinear—a separate brood identity is assigned only when a female was observed in multiple years—brood identity was not evaluated in these variance-component tests. To this end, we performed a variance component test, which compared the log-likelihoods of models with and without the random effect (varCompTest function of the “varTestnlme’ package in R; Baey and Kuhn 2023). Likelihood ratio tests showed no evidence of meaningful random variation for either female identity or year (all *P* ≥ 0.45), with variance components estimated as zero. Thus, standard logistic regression, rather than mixed-model logistic regression, was appropriate for analyzing the data.

Model selection was carried out with the MuMIn package (Bartoń 2020) comparing the AICc with all permutations of variables, with a few notable exceptions. First, to avoid overfitting—particularly since binomial logistic regression is sensitive to over-parameterization—we excluded alternative indicators of parental quality (age, body condition and relative head size) from the same models. Thus, relative head size and body condition were moderately correlated (Pearson's r = 0.404, *P* < 0.001, *n* = 233) and are both size-corrected residuals using radius-ulna length, making them partly non-independent; preliminary models that included both predictors produced unstable coefficients and loss of significance consistent with over-parameterization, underscoring why these traits were modeled separately. Second, viable brood size was forced into all candidate models in the second analysis, to accommodate the expected reduction in the odds of at least one duckling leaving the natal brood with an increase in brood size. To derive robust estimates of parameter values and variable importance, we averaged all models with ΔAIC < 2 relative to the best-fitting model (‘model.avg’ function, MuMIn package) following the approach of Burnham and Anderson (2002). Two top models within 2 ΔAICc were identified in both analyses (Table S1). Conditional model averaging was applied because of the inclusion of a priori forced covariates and/or the exclusion of certain variable combinations (see above). Collinearity among variables in the final selected models was not detected, as all variance inflation factors (VIFs) were below the conservative threshold of 3 (Zuur et al. 2009). Goodness-of-fit of final models was assessed using Nagelkerke's pseudo *r*^2^ (Nagelkerke 1991). Effect sizes were determined by converting Nagelkerke's pseudo *r*^2^ to Cohen's *f*^2^ using the formula *r*^2^/(1 − *r*^2^), where *f*^2^ values of 0.02, 0.15 and 0.35 correspond to low, medium and large effects, respectively (Cohen 1988).

## Results

**Fig. 1.**
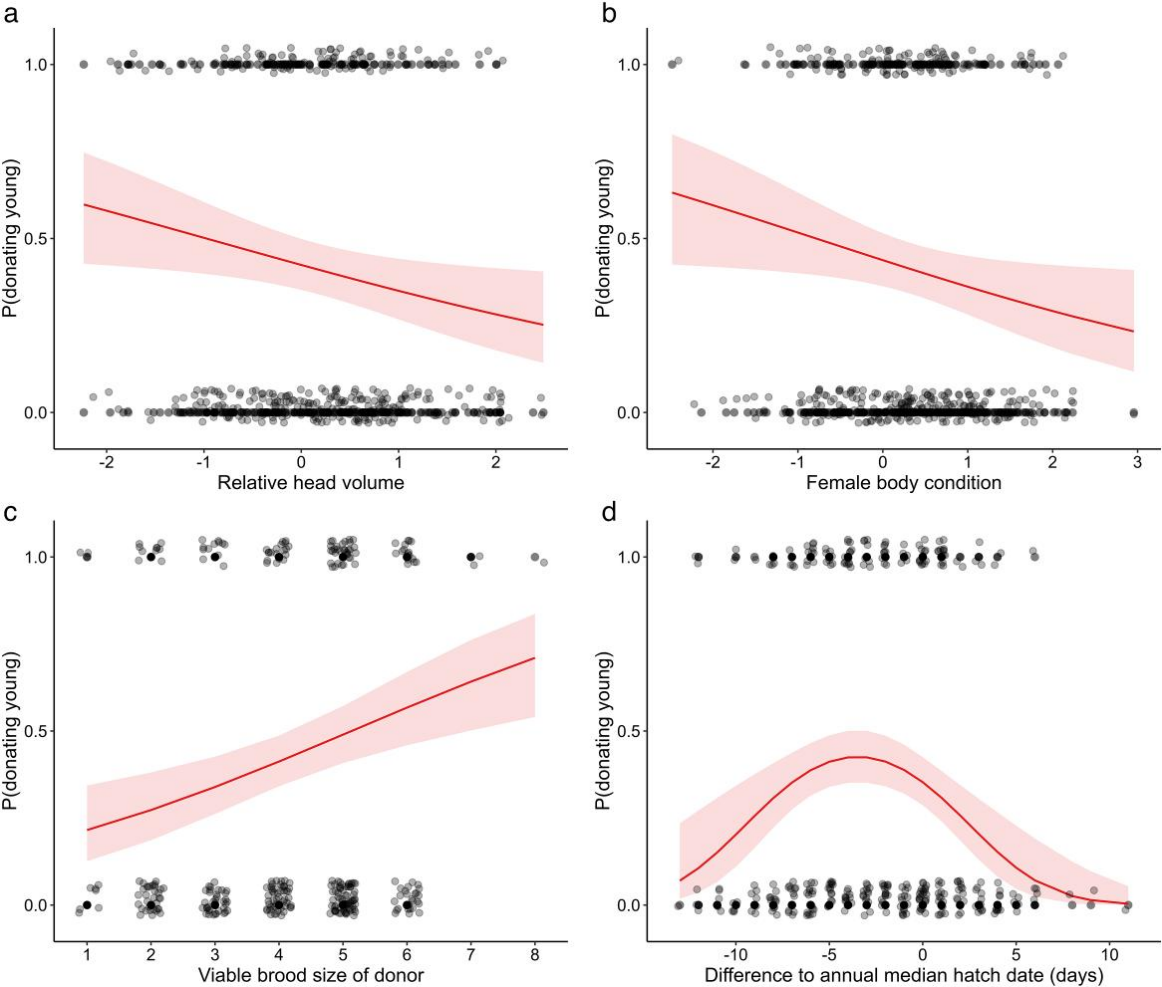
The odds of a female common eider donating/losing at least one offspring to other broods in relation to her relative head size (a), body condition at hatching (b), viable brood size at hatching (c) and the deviation between the female's hatch date and the median annual hatch date (d). Solid regression lines indicate significant relationships, and light red areas account for 95% confidence intervals.

Out of 343 observations, 119 (34.7%) concerned cases in which at least one offspring was permanently adopted into another brood. After model averaging, the significant explanatory variables of female status as an offspring “donor’ were female relative head size and body condition, viable brood size at hatching, and the linear and quadratic difference between the focal female's hatch date and the median annual hatch date (Table 1). Larger relative head size (Fig. 1a) and better body condition at hatching (Fig. 1b) were associated with lower odds of a female donating (losing) at least one offspring to other broods. In contrast, an increase in viable brood size at hatching (Fig. 1c) was associated with higher odds of donating offspring. Furthermore, the odds of being an offspring donor peaked just prior to the hatching peak in the population, with the odds being lower at both ends of the breeding season (Fig. 1d), reflected in the significant quadratic term of this variable (Table 1). The Nagelkerke pseudo *r*^2^ for the two top-ranked models (Table S1) were 0.126 and 0.124, respectively, representing a small-to-medium effect size (*f*^2^ = 0.144 and 0.142, respectively).

**Table 1.** Model-averaged parameter estimates and standard errors (E ± SE), z-values (z) and *P*-values (*P*) derived from the two top-ranked GLMs (ΔAICc ≤ 2) explaining status of female common eiders as “donors” of young (at least one offspring transferred) in relation to intrinsic attributes (relative head size and body condition at hatching), viable brood size at hatching, and the availability of potential recipients (deviation from median hatching and its quadratic effect).

Parameter	E ± SE	z	*P*
**Intercept**	**−1.812** ± **0.415**	**4.35**	**<0.001**
**Relative head size**	**−0.314** **±** **0.135**	**2.312**	**0.021**
**Body condition**	**−0.319** **±** **0.146**	**2.179**	**0.029**
**Viable brood size**	**0.316** **±** **0.091**	**3.478**	**<0.001**
**Deviation from hatch median**	**−0.183** **±** **0.055**	**3.326**	**<0.001**
**Deviation from hatch median^2^**	**−0.0257** **±** **0.007**	**3.493**	**<0.001**

Significant effects (*P*-value ≤ 0.05) are presented in bold.

**Fig. 2.**
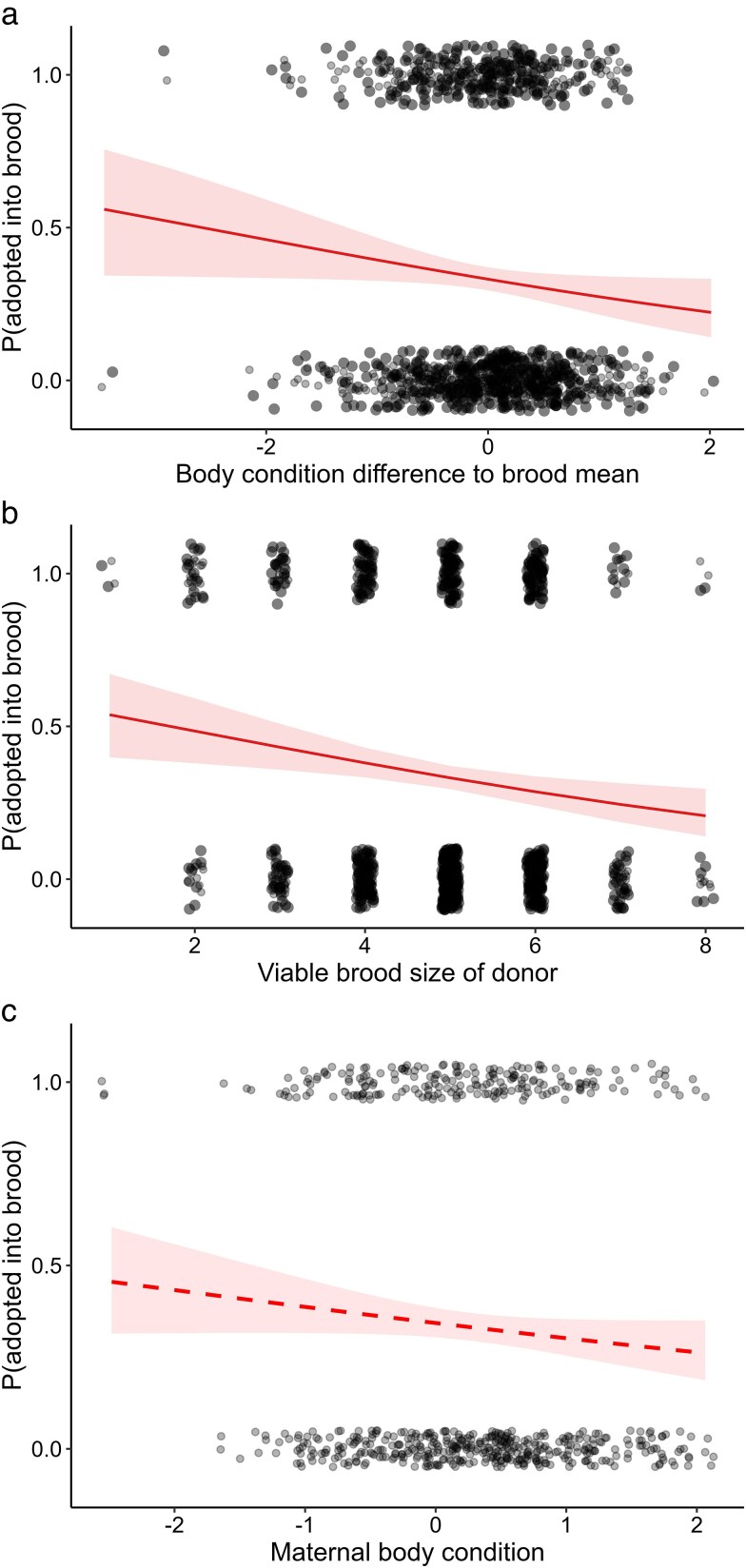
The odds of a common eider duckling being permanently adopted into another brood after nest exodus in relation to body condition relative to its natal brood mates (a), viable brood size at hatching (b) and maternal body condition at hatching (c). Solid and dashed regression lines indicate significant and marginally significant relationships, respectively, and light red areas account for 95% confidence intervals.

The best-performing single-predictor model of whether a duckling stayed or left its natal brood was brood-mean-centered duckling body condition at hatching, rather than body weight (ΔAICc = 0.628) or tarsus length (ΔAICc = 3.95). After model averaging, the significant explanatory variables of duckling transfer status included brood-mean-centered duckling body condition, viable brood size at hatching, and maternal body condition (Table 2). The odds of a duckling being adopted to another brood were higher for a duckling in poorer body condition than its natal brood mates (Fig. 2a). Viable brood size showed a negative relationship with adoption odds (Fig. 2b), an outcome expected from the analysis inclusion criterion (only broods with at least one adoption were considered), which inherently reduces the per-duckling probability of adoption as brood size increases (see Statistical analysis). We also found that higher body condition of the natal (donor) female was marginally associated with lower odds that a duckling was adopted into another brood (Fig. 2c, Table 2). The Nagelkerke pseudo *r*^2^ for the two top-ranked models (Table S1) were 0.037 and 0.0304, respectively, corresponding to a small effect size (*f*^2^ = 0.039 and 0.031, respectively).

**Table 2.** Model-averaged parameter estimates and standard errors (E ± SE), z-values (z) and *P*-values (*P*) derived from the two top-ranked GLMs (ΔAICc ≤ 2) explaining whether a common eider duckling was permanently adopted into another brood in relation to its brood-mean-centered duckling body condition at hatching, viable brood size at hatching, and maternal body condition at hatching.

Parameter	E ± SE	z	*P*
Intercept	0.421 ± 0.353	1.189	0.234
**Duckling body condition**	**−0.276** **±** **0.130**	**2**.**115**	**0.034**
**Viable brood size**	**−0.218** **±** **0.071**	**3.051**	**0.002**
Maternal Body Condition	−0.190 ± 0.108	1.75	0.080

Significant effects (*P*-value ≤ 0.05) are presented in bold.

## Discussion

We examined associations between parental and offspring traits and the likelihood of offspring transfer. Donor traits linked to individual quality—large relative head size and good body condition—were associated with reduced odds of losing offspring to other broods. Similarly, high-quality offspring in good body condition relative to their brood mates were more likely to remain in their natal brood. These results are consistent with selection for maternal traits that preserve brood integrity and for offspring traits that prevent separation from the natal brood. Female age was not associated with offspring donation; this result held whether age was treated as continuous variable (as done here) or as a dichotomy (first-time breeder vs. previously ringed). Nevertheless, because our observational design cannot determine whether the proximate mechanisms and constraints associated with offspring donation are adaptive (fitness-enhancing), we next assess evidence on both sides, highlighting the complementarity of proximate and ultimate explanations. Given that post-hatch brood amalgamation (ie, the mixing of broods and individual young after hatching) may result from accidental brood mixing (Munro and Bédard 1977)—a proximate manifestation of the broader “reproductive-error” or misdirected-care hypotheses (Andersson and Eriksson 1982; Riedman 1982; Brown 1998)—and constraints on donors and prospective adoptees (eg, this study), it is worth asking why it is so common (Eadie et al. 1988). However, acceptance of non-natal offspring may benefit recipients, not only through passive mechanisms such as predator dilution (Jaatinen et al. 2011), but also through nepotistic parental behavior (see below), which is difficult to reconcile with the accidental-mixing hypothesis. It is nevertheless important to acknowledge that particularly the analysis of duckling adoptee status had modest statistical power, most likely because of a complex set of unmeasured confounders such as variation in ambient weather conditions and chance predation events (Traylor et al. 2008). Alternatively, but not exclusively, ducklings in poorer condition, particularly when born to mothers in poor condition, may simply be more likely to be lost (via abandonment or rapid predation); because untended ducklings are quickly killed by predators, our observed adoptee sample may underrepresent the total pool of lost ducklings.

### Factors associated with female status as “donor’ of offspring

While acknowledging the limits of observational inference, it is noteworthy that maternal traits typically associated with higher individual quality—larger relative brain size and better body condition—were linked to a lower probability of donating offspring (Fig. 1a and b). Adoptees have often been reported to show reduced survival relative to offspring belonging to the recipient female, or to offspring remaining with their mother (Bustnes and Erikstad 1991; Nastase and Sherry 1997; Öst and Bäck 2003), but direct tests comparing adoptee survival with that of ducklings remaining with the natal female are rare. In one of the few such studies, Eadie and Lyon (1998) found no significant difference in the Barrow's goldeneye (*Bucephala islandica*). Donation could in principle also benefit donors if a reduced brood size improved maternal survival or future reproduction, yet studies in eiders provide little support for such benefits: antipredator vigilance by brood-tending females is unrelated to brood size (Öst et al. 2007a), and survival may be higher, not lower, for females laying larger clutches (Yoccoz et al. 2002). Although our analyses focus on donors and transferred offspring (recipients could not be unambiguously identified in multi-tender broods; see Methods), also the recipient perspective is important because recipient costs and benefits influence the interpretation of potential costs to donors. From the recipient's perspective, any costs of caring for additional young are substantially reduced and even negated in this system. Thus, a larger brood size is not expected to influence antipredator vigilance in brood-tending female eiders (Öst et al. 2007a), and per capita duckling survival increases with clutch size (Öst et al. 2008a), as is common for many waterfowl (Andersson 2017). Importantly, adopted young occupy more peripheral positions within broods, where their presence correlates with increased aggression by host females (Öst and Bäck 2003). Predation in broods is edge-biased (Swennen 1989), aligning with the concept of marginal predation (Hamilton 1971), a theory well-supported by empirical evidence (Stankowich 2003; Hirsch and Morrell 2011; McDowall and Lynch 2019).

Kin and sibling recognition is considered adaptive in species with frequent mixing of dependent young (Nakagawa and Waas 2004). While cognitive constraints may be associated with the failure to discriminate parasitic eggs or offspring (eg, Rothstein 1978; Shizuka and Lyon 2010), they are rarely considered in the context of potential “donors’ maintaining brood integrity. Errors in parent-offspring recognition per se (‘reproductive error’ hypothesis; Andersson and Eriksson 1982; Riedman 1982) are unlikely to be the main cause of mother-offspring bond disintegration. Thus, female eiders maintain close spatial association with their own ducklings; adopted ducklings tend to occupy more peripheral, presumably more dangerous, positions; and females increase aggression in the presence of non-natal young (Öst and Bäck 2003). Furthermore, females adjust parental effort (antipredator vigilance) in relation to their share of offspring in amalgamated broods (Öst et al. 2007a)—taken together, these findings, albeit correlational, are inconsistent with recognition errors. Instead, failure to maintain brood cohesion may be a more likely mechanism explaining why relatively small-brained females faced an increased risk of mother-offspring bond disintegration. Such failures may be due to limited maternal attention or compromised numerical abilities—linked to reduced domain-general cognitive capacity (eg, Bryer et al. 2022)—perhaps reflected in a difficulty to assess and ensure the completeness of the brood. However, whether numerical competence relates to individual variation in (relative) brain size is currently unknown and should be addressed in future studies.

We found that females in poor body condition were more likely to be donors of offspring, echoing previous studies in eiders (Bustnes and Erikstad 1991; Kilpi et al. 2001) and related species (Traylor et al. 2008). Our results are consistent with the notion that nutritionally challenged females actively abandon offspring as a salvage strategy (Eadie et al. 1988). However, they are equally compatible with an alternative in which condition-dependent parental investment and grouping decisions (larger coalitions, reduced individual vigilance) increase passive separation of offspring. Thus, female eiders in good body condition are likely to make a greater investment in parental care, as their elevated prolactin levels (Mohring et al. 2024) may hormonally prime them for such commitment (Angelier et al. 2016). Enhanced parental behavior may manifest as increased antipredator vigilance by mothers in good condition (Öst et al. 2007a) and more active brood defense, as prolactin is linked to a stronger reluctance to leave the brood unattended during disturbances (Pedersen 1989; Wang and Buntin 1999). Such increased investment in parental care should reduce the likelihood of offspring becoming separated from their original brood. Furthermore, females in poorer body condition are found in larger brood-rearing coalitions (Öst et al. 2003b), which reduces the cost of care as division of labor among group members allows females to reduce individual vigilance and increase time spent foraging (Öst et al. 2002). However, as a side effect, larger duckling aggregations may predispose ducklings to become separated from their parents due to a more dispersed spatial arrangement (Morrell and Romey 2008). Thus, while our findings are consistent with both the salvage strategy hypothesis for brood abandonment and the alternative explanation of passive separation through condition-dependent care and grouping, the present data do not allow us to distinguish between these mechanisms.

Optimal parental-investment theory predicts increased care as the relative value of current reproduction increases (Trivers 1972), and female antipredator vigilance does increase with the ratio of a female's brood to total ducklings in the brood amalgamation (Öst et al. 2007a). Nevertheless, females with larger broods at hatching were more likely to donate offspring (Fig. 1c); together with the lower loss of young by relatively large-headed or better-condition females, this pattern points to constraints—perhaps because larger broods tend to be more spatially dispersed, compromising brood integrity (Morrell and Romey 2008). Interestingly, Eadie and Lyon (1998) report the opposite pattern for brood desertion in Barrow's goldeneyes, showing experimentally that females were more likely to desert broods that had been reduced to small sizes, ie, the likelihood of desertion increased as brood size decreased. However, parental desertion may entail an active, often abrupt brood-level decision (affecting the entire brood at once), whereas our finding concerns the probability of donating or losing individual offspring, which increases with brood size, likely driven by statistical effects (more ducklings mean more chances for loss) and brood-cohesion constraints in larger groups.

The relationship between the odds of being an offspring donor and relative hatching date was curvilinear and peaked shortly prior to the hatching peak in the population. This result likely reflects the combined outcome of at least three factors: seasonal variation in potential recipient availability—approximated by the difference between each female's hatch date and the median annual hatch date (Jaatinen and Öst 2013), the tendency of similar-sized and -aged broods to mix more readily (Savard et al. 1998; Öst and Bäck 2003; Öst et al. 2003a; Jaatinen and Öst 2013), and the presence of a time window after which the opportunity for brood mixing declines or is lost completely (Eadie and Lyon 1998). While accepting offspring larger than the recipient's own young imposes reproductive costs, the opposite scenario—why smaller adoptees are rarely accepted—is more challenging to explain. However, the presence of smaller offspring in the brood could reduce the rate of movement (Anderson and Alisauskas 2001) and potentially delay the diet shift from near-shore food items of small ducklings (particularly gammarids, *Gammarus* spp.) to those preferred by larger ducklings and occurring farther offshore (blue mussels, *Mytilus* spp.; Gorman and Milne 1972; Öst and Kilpi 1999). Furthermore, since females remain with young until they reach a certain developmental stage (Öst et al. 2003a), the presence of smaller or younger adoptees in the foster brood could prolong this period and thereby increase parental care costs (Eadie and Lyon 1998). It is important to note that the relationship between hatching date and donorship is asymmetric around the median hatching date, with donorship being relatively more common before than after peak hatching (Fig. 1d). As an illustrative example, the predicted probability of a female acting as a donor of young if the female hatched her brood five days before or after the median hatching date was 0.42 and 0.11, respectively (other explanatory variables held at their means). This likely reflects the more uniform age distribution of ducklings during the early brood-rearing season, which better facilitates adoption compared to the late season (Traylor et al. 2008).

### Factors associated with duckling status as adoptee

To briefly recap the prediction: if all ducklings are equally attracted to broody or dominant females, higher-quality ducklings may be overrepresented among adoptees because of better post-adoption survival—consistent with evidence that relative duckling body condition predicts later recruitment in eiders (Christensen 1999, 2002) and with observations in gulls that larger adoptees can realize higher survival at the expense of the foster parent's siblings (Brown 1998). In contrast, if the split of mother–offspring bonds is associated with physical constraints, an excess of lower-quality adoptees is expected. Consistent with the latter physical-constraint scenario, we found that ducklings that were adopted were in poorer body condition than their counterparts remaining with their mother. In terms of proximate mechanisms, individuals with greater food requirements—ducklings that are in poorer conditions than their natal siblings—are more likely to wander off from the brood in search of food due to their greater incentive to feed (Swennen 1989; Romey 1996). Conversely, increased levels of satiation are expected to increase the preference for central positions in groups (Morrell and Romey 2008). Furthermore, well-fed ducklings are more likely to remain close to their mothers during predation attempts or conspecific aggressive encounters owing to faster escape responses (Swennen 1989; Anderson and Alisauskas 2001). Based on these results, we cannot rule out the possibility that adoption seeking may represent an alternative tactic adopted to mitigate the effects of inadequate parental care, as has been argued for geese (Kalmbach 2006) and larids (Saino et al. 1994; Brown 1998). Nevertheless, unlike the more dependent young of geese and larids, eider ducklings are precocial and largely self-sustaining from hatch, which reduces the scope for extended parental provisioning and makes adoption-seeking as a compensatory tactic less plausible. Conversely to the tendency of poor-quality ducklings to be separated from the mother more often, our study reveals a novel mechanism favoring individuals in relatively good condition: their greater success at remaining in the natal brood may enhance survival and recruitment by reducing exposure to nepotistic alloparental behavior (Öst and Bäck 2003).

Increasing viable natal brood size was associated with lower odds of being adopted into a brood. No doubt this partly reflects the fact that only broods with at least one adopted offspring were included in the analysis. However, methodological considerations apart, pairs with smaller broods may more often suffer total brood loss to adoption (Zicus 1981). This effect could occur through several non-exclusive mechanisms. First, parents are expected to respond to cues of reduced brood value—be it small brood size (Eadie and Lyon 1998) or low brood success (Pöysä et al. 1997)—by reducing parental investment (Trivers 1972; Carlisle 1982). Second, females with a small brood size have the most to gain from predator dilution in merged broods (Jaatinen and Öst 2013). Group members with larger shares of offspring are expected to more strongly oppose their entry, preventing them from finding willing coalition partners (Jaatinen and Öst 2013). Third, female dominance may be positively correlated with brood size (Loonen et al. 1999) and act as an attractant to ducklings (Munro and Bédard 1977; Kalmbach et al. 2005), and thus the offspring of dominants may less likely end up being adopted. However, we consider this explanation less likely because female dominance in eider brood-rearing coalitions is maintained through age-dependent agonistic interactions (Öst et al. 2007b), and female age was neither related to the odds of females being donors nor ducklings becoming adoptees. To conclude, both reduced parental investment and brood-size dependent acceptance into brood-rearing coalitions may be associated with the reduced chance of offspring “donation’ in small broods.

We found that ducklings tended to more often be adopted if their original mother was in lower body condition, a finding that was marginally significant. A plausible explanation is that maternal condition may be linked to both maternal behavior and the within-brood condition asymmetry of ducklings. First, vigilance effort is positively related to maternal body condition in eiders (Öst et al. 2007a), which should facilitate keeping track of the brood. Second, egg size asymmetry within clutches increases with decreasing maternal body condition in eiders (Hanssen et al. 2002), and egg size largely determines hatching body weight (Erikstad et al. 1998) and condition (Öst et al. 2020). Importantly, duckling body condition was centered within broods, reflecting each duckling's relative condition rather than absolute brood quality. The greater expected condition asymmetry in broods tended by poor-condition females, coupled with the fact that ducklings in relatively poor condition are more inclined to become adopted (Fig. 2a), should create favorable conditions for finding stragglers at risk of being separated from their poor-condition mother.

### Conclusions

We analyzed ten years of data on eiders to investigate how maternal and offspring characteristics affect, on the one hand, the likelihood of acting as an offspring donor and, on the other hand, the likelihood of a duckling becoming adopted into another brood. Presumed maternal fitness proxies—good body condition and large relative brain size—were associated with greater ability to preserve brood integrity. Correspondingly, better-quality offspring—those in better body condition than their siblings—were more likely to remain with their mother. Although brood amalgamation might provide fitness benefits to low-quality donors and the transferred offspring, the low post-hatch parental care costs—food-guidance, brooding, and protection rather than direct feeding—suggest such gains may be limited; we therefore treat adaptive interpretations cautiously. Because brood amalgamation and offspring transfer between broods are widespread in waterfowl, the dynamics of brood amalgamation observed here—in a system with precocial young, low parental care demands and high predation risk that favors larger brood aggregations—are likely relevant to many Anseriform species. However, future work directly quantifying the fitness costs and benefits for all parties involved under different environmental contexts, including fine-scale variation in weather conditions and predation risk (cf. Traylor et al. 2008), is necessary to further understand the drivers of brood amalgamation in this and other species alike.

## Supplementary Material

araf134_Supplementary_Data

## Data Availability

Analyses reported in this article can be reproduced using the data provided by Öst et al. (2025).
