# Supplementary material for: Breaking bonds: maternal and offspring states relate to constraint-based brood donation in a seaduck
Source: Behav Ecol. 2025 Nov 21;37(1):araf134. doi: 10.1093/beheco/araf134 (PMC12701309; doi:10.1093/beheco/araf134)
Supplement: araf134_Supplementary_Data [file araf134_supplementary_data.doc]

Supplementary data for

**Breaking bonds: maternal and offspring states relate to constraint-based brood donation in a seaduck**

**Supplementary Table S1**: Top-ranked (ΔAICc ≤ 2) binomial general linear models explaining (1) female status as offspring ‘donors’ (1: at least one offspring transferred, 0: no transfer), (2) duckling status as adoptees (1) or remaining in their natal brood (0). Explanatory variables: maternal relative head size (RHS) and body condition at hatching (MBC), viable brood size at hatching (VBC), the linear (HD) and quadratic difference (HD^2^) between the focal female’s hatch date and the median annual hatch date (analysis 1), and brood-mean-centered duckling body condition at hatching (DBC; analysis 2). Female quality attributes (RHS and MBC) were not included in the same models and viable brood size was a forced covariate in the second analysis (see text).

| **Analysis** | **Model** | **df^1^** | **log-lik^2^** | **AICc** | **ΔAICc** | ***w*_i_^3^** | **R^2^m^4^** |
| --- | --- | --- | --- | --- | --- | --- | --- |
| Female donor status | VBS + RHS + HD + HD^2^ | 5 | -205.01 | 420.19 | 0.00 | 0.58 | 0.13 |
|  | VBS + HD + HD^2^+ MBC | 5 | -205.31 | 420.81 | 0.62 | 0.42 | 0.12 |
| Duckling adoptee status | DBC + VBS + MBC | 4 | -387.73 | 783.50 | 0.00 | 0.63 | 0.04 |
|  | DBC + VBS | 3 | -389.28 | 784.60 | 1.07 | 0.37 | 0.03 |

Models were compared using Akaike Information Criteria for small sample sizes (AICc) and ranked using Akaike distances (ΔAICc).

^1^ df: degrees of freedom

^2^ log-lik: log-likelihood

^3^ *w*_i_: Akaike weight

^4^ R^2^m: Nagelkerke’s pseudo R^2^ of model
